# Association between dietary vitamin B6 intake and endometriosis risk: evidence from the national health and nutrition examination survey

**DOI:** 10.3389/fnut.2024.1407099

**Published:** 2024-10-03

**Authors:** Ling Yin, Feng Liang, Baoli Xie, Yanlin Su, Li Cheng, Xin Wei, Wencai Tian

**Affiliations:** ^1^Department of Obstetrics and Gynecology, The Affiliated Changsha Central Hospital, Hengyang Medical School, University of South China, Changsha, China; ^2^Gynecology Department, The Reproductive Hospital of Guangxi Zhuang Autonomous Region, Nanning, China; ^3^Gynecology Department, The First People’s Hospital of Nanning, Nanning, China

**Keywords:** endometriosis, vitamin B6, NHANES, cross-sectional study, dietary intake

## Abstract

**Background:**

Endometriosis is a multifaceted disorder with genetic, immune, inflammatory, and multifactorial origins. Vitamin B6 serves as a pivotal coenzyme in various metabolic pathways involving lipids, hemes, nucleic acids, proteins, and carbohydrates. Dysregulation or deficiency of vitamin B6 can perturb human physiology. However, the relationship between dietary vitamin B6 and endometriosis remains elusive. This study aims to explore how dietary intake of vitamin B6 is associated with the risk of endometriosis.

**Methods:**

Using cross-sectional data from the National Health and Nutrition Examination Survey, we analyzed information from American women aged 20–54 years between 1999 and 2006. After adjusting for relevant covariates, multivariable logistic regression analysis was employed to evaluate correlations.

**Results:**

A total of 4,453 women were included in the study. The multiple linear regression model revealed a positive association between dietary vitamin B6 intake and the risk of endometriosis, even after controlling for confounding variables. Compared to individuals with lower vitamin B6 consumption (Q1: <0.94 mg/day), the adjusted odds ratio (OR) values for dietary vitamin B6 intake and endometriosis in Q2 (0.95–1.39 mg/day), Q3 (1.40–1.99 mg/day), and Q4 (>1.90 mg/day) were 1.22 (95% CI: 0.88–1.69, *p* = 0.23), 1.22 (95% CI: 0.86–1.73, *p* = 0. 279), and 1.51 (95% CI, 1.01–2. 24, *p* = 0.04), respectively.

**Conclusion:**

Our findings suggest a positive correlation between endometriosis and dietary vitamin B6 intake. Further investigations are imperative to establish a causal relationship between dietary vitamin B6 intake and endometriosis.

## Introduction

Endometriosis is an inflammatory condition characterized by the ectopic growth of endometrial-like tissue outside the uterus, often affecting pelvic organs (1). It affects around 176 million women globally, leading to symptoms such as pelvic pain and infertility in 5–10% of cases (2). Those affected typically incur double the healthcare costs compared to unaffected individuals (3), making it a significant public health concern (4). Despite its impact, there is limited understanding of modifiable risk factors associated with its development.

Vitamin B6, a water-soluble vitamin found naturally in various foods, is involved in numerous metabolic processes as a coenzyme (5). It exists in three forms: pyridoxamine, pyridoxal, and pyridoxine, with pyridoxal-phosphate (PLP) being the active form. Vitamin B6 also plays a role in the immune and endocrine systems and has antioxidant properties (6, 7). Studies have suggested that vitamin B6 deficiency may increase the risk of various cancers, while adequate levels might reduce the risk, although findings are mixed (8–13). There is also evidence linking high doses of vitamin B6 with bone issues and other health concerns (14–16). Despite these associations, its potential protective effects against conditions like depression, cardiovascular disease, and cognitive decline remain unconfirmed (17–19).

The biological behaviors of endometriosis and malignancies, particularly in pathways regulating inflammation and cell proliferation, indicate a potential role for vitamin B6 in modulating these processes (20). Limited research has explored the relationship between dietary vitamin B6 and endometriosis. One study reported lower vitamin B6 levels in patients with advanced endometriosis (21). This study seeks to investigate the association between dietary vitamin B6 intake and endometriosis in a large sample of American women aged 20 to 54, aiming to offer new insights into potential preventive strategies.

## Methods

### Data source

The National Health and Nutrition Examination Survey (NHANES) and the National Center for Health Statistics (NCHS) played essential roles in collecting data for this investigation. We acquired data from three consecutive 2-year NHANES cycles conducted between 1999 and 2006, utilizing a nationally representative stratified sample through interviews and physical examinations. The NCHS Ethics Review Committee granted ethical approval, and all subjects provided signed informed consent.

### Study design and population

**Figure 1:**
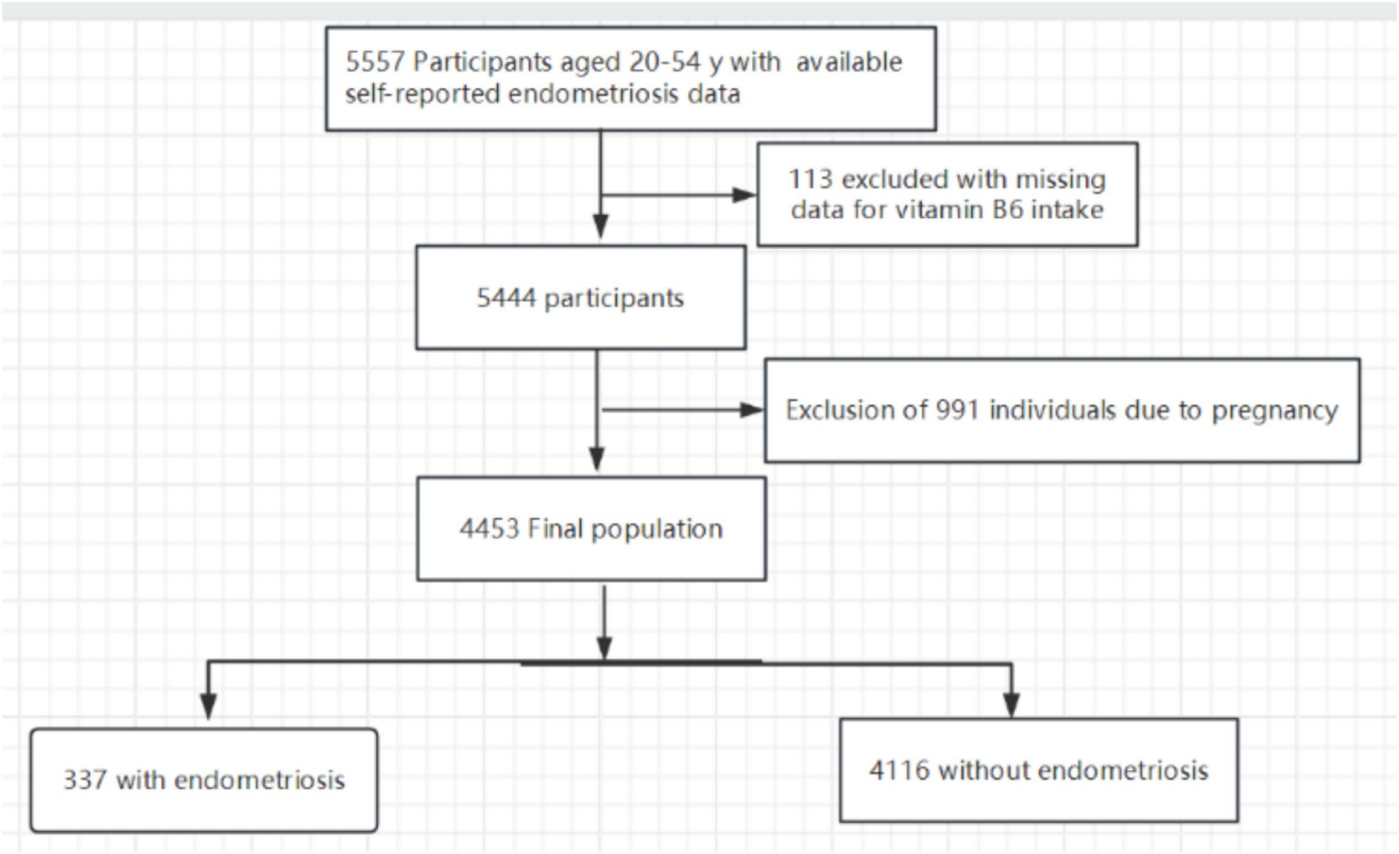
The study’s flow diagram.

The original dataset included 5,557 female participants. After excluding individuals with missing endometriosis-related information and those outside the age range of 20 to 54 years, our final study population comprised 4,453 women. Among them, 337 were diagnosed with endometriosis, while 4,116 were not. Exclusions were made for 991 pregnant women and 113 individuals lacking information on dietary vitamin B6 intake. Figure 1 provides a detailed illustration of the inclusion and exclusion process.

### Participants and definition

We determined endometriosis based on participants’ responses to a specific question in the reproductive health questionnaire: “Has a doctor or other health professional ever diagnosed you with endometriosis?” We categorized those who answered affirmatively as patients. Professional interviewers assessed dietary vitamin B6 consumption during the NHANES dietary survey, a component of the “What We Eat in America” survey, conducted at the Mobile Examination Center (MEC) using a 24-h recall method. The NHANES computer-assisted dietary interview (CADI) system recorded participants’ food and beverage intake from the 24 h preceding the interview.

According to the study procedure, we randomly assigned participants to data collection sessions that occurred in the morning, afternoon, or evening. We determined dietary vitamin B6 and nutrient consumption using the US Department of Agriculture Survey Nutrients Database and the University of Texas Food Intake Analysis System. We excluded pharmaceuticals and dietary supplements from the nutritional calculations. We conducted two 24-h dietary recall interviews, followed by a phone interview 3 to 10 days later. We selected the first interview, conducted in person at the MEC, for analysis, as the 24-h recall method is the most commonly used in large-scale surveys (22).

### Measurements

In our study, we considered a wide range of covariates sourced from the literature (23–25), including Age, marital status, race/ethnicity, education level, family income, smoking status, physical activity, BMI, alcohol consumption, use of birth control pills, high blood pressure, diabetes, coronary heart disease, chronic bronchitis, caloric consumption, total fat intake, total cholesterol intake, and usage of nutritional supplements. We categorized race and ethnicity into Non-Hispanic White, non-Hispanic Black, Mexican American, and other races. We classified marital status as either living with a partner or living alone. We stratified educational attainment into three levels: fewer than 9, 9 to 12 years, and more than 12 years. We assessed family income using the Poverty Income Ratio (PIR) and categorized it into low, medium, and high income, based on ranges from 1.3 to 3.5, according to the US government’s Agriculture report (26). We dichotomized smoking status into smokers and never smokers (those who have smoked fewer than 100 cigarettes in total). Alcohol drinking status was determined by the survey question, “In any 1 year, have you had at least 12 drinks of any type of alcoholic beverage?” Participants who answered “yes” were defined as alcohol drinkers. We divided physical activity levels into three categories: unable to perform physical activity, moderate (defined as at least 10 min of movement within the previous 30 days resulting in light perspiration or a mild to moderate increase in respiration or heart rate), and vigorous (at least 10 min of activity within the last 30 days resulting in profuse sweating or an increased heart rate). The determination of previous disease (high blood pressure, diabetes, coronary heart disease and chronic bronchitis) was based on the inquiry in the questionnaire of whether the doctor had been informed of the condition in the past.

Prior to the MEC interview, participants completed a food recall questionnaire in order to get 24-h nutritional data, including macronutrient profiles and calorie intake. Additionally, details regarding medications, including dietary supplements consumed within the preceding month, were documented.

### Statistical analyses

In this study, we conducted a secondary analysis of publicly available datasets and used descriptive statistics to characterize continuous variables (mean/SD or median/IQR) and proportions (%) for categorical variables. We assessed group differences using Kruskal-Wallis tests and one-way analyses of variance. We employed logistic regression models to explore the relationship between dietary vitamin B6 intake and endometriosis across three models. Model 1 adjusted for sociodemographic (age, race/ethnicity, education level, family income and marital status); Model 2 was adjusted for Model 1 plus BMI, smoking status, vigorous activity, moderate activity, alcohol consumption, birth control pills taken, high blood pressure, diabetes, coronary heart disease and chronic bronchitis; and Model 3 was adjusted for Model 2 plus calorie consumption, total fat consumption, total cholesterol consumption and dietary supplements taken. These models aimed to comprehensively address potential confounding factors and to enhance our understanding of the relationship between dietary vitamin B6 intake and endometriosis.

Furthermore, we investigated potential modifiers of the association between dietary vitamin B6 intake and endometriosis by incorporating variables such as family income (low vs. medium or high), marital status (living with a partner vs. living alone), smoking status, and dietary supplements taken. We assessed heterogeneity among subgroups using multivariate logistic regression and examined interactions between subgroups and dietary vitamin B6 intake through likelihood ratio testing. To ensure the robustness of our findings, we conducted sensitivity analyses by excluding participants with extreme energy intake, defined as consuming less than 500 or more than 5,000 kcal per day. This meticulous approach aimed to assess the consistency and reliability of our results under different conditions, thereby enhancing the validity of the study outcomes.

We determined the sample size based on available data, without conducting *a priori* statistical power assessments. We performed statistical analyses using R 3.3.2 and Free Statistics Software 1.5 (27). We conducted a comprehensive descriptive study on all individuals. For hypothesis testing, we utilized a two-tailed analysis, considering a significance level of 0.05 for determining statistical significance. This widely accepted threshold ensures standard confidence levels for interpreting results and drawing meaningful conclusions from the analyses.

## Results

### Baseline characteristics

Table 1 presents the essential characteristics of the 4,453 participants in the study. Among the sample, 337 individuals (7. 57%) received a diagnosis of endometriosis, and 2,820 (63.92%) were classified as overweight. A total of 1741 participants (39.10%) reported being smokers, while 2099 (47.14%) acknowledged the use of dietary supplements. Regarding age distribution, 2,378 participants (53.40%) were below 40 years old, 1,424 (31.98%) were between 40 and 50 years old, and 651 (14.62%) were above 50 years old. Notably, dietary vitamin B6 intake appeared higher in individuals who used dietary supplements, engaged in moderate exercise, were aged under 40, had a BMI below 25, had an education level exceeding 12 years, did not smoke, and lived with a partner or had a higher income. These baseline characteristics provide an overview of the diversity within the study population and establish the foundation for subsequent analyses exploring the association between these factors and endometriosis.

**Table 1:** Population characteristics by categories of dietary vitamin B6 intake.

			Dietary vitamin B6 intake(mg/d)			
Variables	Total	Q1(<0.938)	Q2(0.939–1.385)	Q3(1.386–1.985)	Q4(>1.985)	*p*-value
No.	4,453	1,106	1,118	1,115	1,114	
Age (year), n (%)						0.355
< 40	2,378 (53.40)	581 (52.53)	593 (53.04)	578 (51.8)	626 (56.19)	
40–50	1,424 (31.98)	365 (33.00)	352 (31.48)	377 (33.81)	330 (29.62)	
> 50	651 (14.62)	160 (14.47)	173 (15.47)	160 (14.35)	158 (14.18)	
BMI (Kg/m^2^), n (%)						0.041
< 25	1,592 (36.08)	360 (32.97)	391 (35.32)	408 (36.89)	1,592 (36.08)	
25–30	1,185 (26.86)	320 (29.30)	313 (28.27)	279 (25.23)	1,185 (26.86)	
> 30	1,635 (37.06)	412 (37.73)	403 (36.40)	419 (37.88)	1,635 (37.06)	
Family Income, n (%)						< 0.001
Low	1,177 (28.28)	346 (33.49)	279 (26.72)	276 (26.54)	276 (26.41)	
Medium	1,521 (36.54)	390 (37.75)	377 (36.11)	385 (37.02)	369 (35.31)	
High	1,464 (35.18)	297 (28.75)	388 (37.16)	379 (36.44)	400 (38.28)	
Race/Ethnicity, n (%)						0.012
Mexican American	1,006 (22.59)	214 (19.35)	263 (23.52)	267 (23.95)	262 (23.52)	
Other Race	400 (8.98)	101 (9.13)	101 (9.03)	109 (9.78)	89 (7.99)	
Non-Hispanic White	2051 (46.06)	501 (45.30)	509 (45.53)	503 (45.11)	538 (48.29)	
Non-Hispanic Black	996 (22.37)	290 (26.22)	245 (21.91)	236 (21.17)	225 (20.20)	
Education Level (year), n (%)						< 0.001
< 9	383 (8.61)	87 (7.88)	97 (8.68)	109 (9.78)	90 (8.09)	
9–12	1,695 (38.10)	488 (44.20)	419 (37.51)	407 (36.50)	381 (34.23)	
> 12	2,371 (53.29)	529 (47.92)	601 (53.80)	599 (53.72)	642 (57.68)	
Family Income, n (%)						< 0.001
Low	1,177 (28.28)	346 (33.49)	279 (26.72)	276 (26.54)	276 (26.41)	
Medium	1,521 (36.54)	390 (37.75)	377 (36.11)	385 (37.02)	369 (35.31)	
High	1,464 (35.18)	297 (28.75)	388 (37.16)	379 (36.44)	400 (38.28)	
Marital Status, n (%)						0.007
Living with a partner	2,613 (60.19)	593 (55.63)	672 (61.37)	680 (62.33)	668 (61.34)	
Living alone	1728 (39.81)	473 (44.37)	423 (38.63)	411 (37.67)	421 (38.66)	
Smoking status, n (%)						< 0.001
Yes	1741 (39.12)	489 (44.21)	431 (38.55)	425 (38.19)	396 (35.58)	
No	2,709 (60.88)	617 (55.79)	687 (61.45)	688 (61.81)	717 (64.42)	
Vigorous activity, n (%)						< 0.001
Yes	1,509 (33.90)	319 (28.84)	364 (32.59)	376 (33.72)	450 (40.43)	
No	2,855 (64.14)	757 (68.44)	734 (65.71)	720 (64.57)	644 (57.86)	
Unable to do activity	87 (1.95)	30 (2.71)	19 (1.70)	19 (1.70)	19 (1.70)	
Moderate activity, n (%)						< 0.001
Yes	2,320 (52.12)	518 (46.84)	569 (50.89)	577 (51.80)	656 (58.94)	
No	2065 (46.39)	566 (51.18)	532 (47.58)	523 (46.95)	444 (39.89)	
Unable to do activity	66 (1.48)	22 (1.99)	17 (1.53)	14 (1.26)	13 (1.17)	
Dietary supplements taken, n (%)						< 0.001
Yes	2099 (47.12)	461 (41.68)	526 (47.05)	522 (46.86)	590 (53.11)	
No	2,350 (52.82)	645 (58.32)	592 (52.95)	592 (53.14)	521 (46.89)	
Diabetes, n (%)						0.608
Yes	236 (5.33)	52 (4.72)	60 (5.42)	62 (5.64)	62 (5.58)	
No	4,176 (93.81)	1,041 (94.12)	1,046 (93.57)	1,043 (93.52)	1,046 (93.89)	
Birth control pills taken, n (%)						0.881
Yes	3,362 (75.52)	825 (74.63)	850 (76.03)	847 (76.01)	840 (75.48)	
No	1,087 (24.43)	279 (25.23)	267 (23.89)	267 (23.93)	274 (24.64)	
Coronary heart disease, n (%)						0.855
Yes	37 (0.81)	10 (0.90)	11 (1.00)	10 (0.92)	6 (0.54)	
No	4,413 (99.12)	1,095 (99.02)	1,107 (99.04)	1,104 (99)0.03	1,107 (99.4.04)	
Chronic bronchitis, n (%)						0.177
Yes	317 (7.12)	90 (8.13)	86 (7.74)	72 (6.45)	69 (6.23)	
No	4,128 (92.67)	1,012 (91.45)	1,032 (92.33)	1,041 (93.24)	1,043 (93.56)	
High blood pressure, n (%)						0.934
Yes	867 (19.45)	213 (19.23)	223 (19.93)	218 (19.62)	213 (19.14)	
No	3,558 (79.92)	885 (80.04)	889 (79.53)	890 (79.82)	894 (80.34)	
Calorie consumption(kcal/d), Mean (SD)	1911.90(816.11)	1332.51(558.03)	1734.22(575.24)	2056.63(651.52)	2520.71(916.92)	< 0.001
Total cholesterol consumption(mg/d), Median (IQR)	194.03 (114.02, 329.03)	117.0 4(71.02, 202.14)	181.03 (113.04, 292.82)	224.04(148.04, 366.45)	272.02 (170.14, 436.83)	< 0.001
Total fat consumption(g/d), Median (IQR)	66.04 (45.40, 91.38)	47.33 (30.43, 65.89)	62.44 (45.45, 82.44)	74.67 (53.43, 99.73)	86.92 (61.64, 117.89)	< 0.001

### Relationship between dietary vitamin B6 intake and endometriosis

Table 2 illustrates the relationships between dietary vitamin B6 intake and endometriosis. Univariate analysis revealed significant associations, indicating correlations between dietary vitamin B6 intake, age, race, family income, education level, use of birth control pills, high blood pressure, chronic bronchitis, and the use of dietary supplements with the presence of endometriosis. These findings underscore a complex interplay between dietary factors, demographic variables, and lifestyle choices influencing the occurrence of endometriosis. Subsequent multivariate analyses will further dissect these associations to elucidate the independent contributions of each factor to the risk of endometriosis.

**Table 2:** Association of covariates and endometriosis risk.

Variable	OR_95CI	P_value	Variable	OR_95CI	P_value
Age(year)			Moderate activity		
< 40	1 (reference)		Yes	1 (reference)	
40–50	0.53 (0.42 ~ 0.68)	<0.001	No	1.21 (0.96 ~ 1.51)	0.108
> 50	0.54 (0.39 ~ 0.74)	<0.001	Unable to do activity	0.49 (0.25 ~ 0.98)	0.043
BMI(Kg/m^2^)			Dietary supplements taken		
< 25	1 (reference)		Yes	1 (reference)	
25–30	0.94 (0.71 ~ 1.24)	0.671	No	1.59 (1.27 ~ 1.99)	<0.001
> 30	1.07 (0.82 ~ 1.39)	0.629	Diabetes		
Family Income			Yes	1 (reference)	
Low	1 (reference)		No	0.76 (0.44 ~ 1.32)	0.33
Medium	0.79 (0.57 ~ 1.08)	0.134	Birth control pills taken		
High	0.51 (0.38 ~ 0.69)	<0.001	Yes	1 (reference)	
Race/Ethnicity			No	2.77 (1.95 ~ 3.93)	<0.001
Mexican American	1 (reference)		Coronary heart disease		
Other Race	0.65 (0.35 ~ 1.19)	0.162	Yes	1 (reference)	
Non-Hispanic White	0.23 (0.15 ~ 0.34)	<0.001	No	1.92 (0.74 ~ 4.96)	0.178
Non-Hispanic Black	0.42 (0.27 ~ 0.67)	<0.001	Chronic bronchitis		
Education Level(year)			Yes	1 (reference)	
< 9	1 (reference)		No	2.67 (1.93 ~ 3.67)	<0.001
9–12	0.19 (0.08 ~ 0.43)	<0.001	High blood pressure		
> 12	0.17 (0.08 ~ 0.39)	<0.001	Yes	1 (reference)	
Marital Status			No	1.61 (1.25 ~ 2.07)	<0.001
Living with a partner	1 (reference)		Calorie consumption(kcal/d)	1 (1 ~ 1)	0.296
Living alone	1.23 (0.97 ~ 1.55)	0.082	Total fat consumption(g/d)	1 (1 ~ 1)	0.651
Vigorous activity			Total cholesterol consumption(mg/d)	1 (1 ~ 1)	0.072
Yes	1 (reference)		Dietary vitamin B6 intake(mg/d)	1.16 (1.02 ~ 1.32)	0.026
No	0.97 (0.77 ~ 1.24)	0.835			
Unable to do activity	0.55 (0.28 ~ 1.06)	0.075			

Compared to individuals with lower vitamin B6 consumption (Q1 < 0.94 mg/day), the adjusted odds ratio (OR) values for dietary vitamin B6 intake and endometriosis in Q2 (0.95–1.39 mg/day), Q3 (1.40–1.99 mg/day), and Q4 (>1.99 mg/day) were 1.22 (95% CI: 0.88–1.69, *p* = 0.24), 1.22 (95% CI: 0.86–1.73, *p* = 0. 279), and 1.51 (95% CI: 1.01–2. 24, *p* = 0.04), respectively (Table 3).

**Table 3:** Association between dietary vitamin B6 intake and endometriosis.

			OR(95%CI)				
Variable	No.	Model 1	*p*-value	Model 2	*p*-value	Model 3	*p*-value
Dietary vitamin B6 intake(mg/d)	4,453	1.19 (1.04 ~ 1.37)	0.011	1.17 (1.02 ~ 1.34)	0.023	1.18 (1.01 ~ 1.39)	0.042
Q1(<0.94)	1,106	1(Ref)		1(Ref)		1(Ref)	
Q2(0.95–1.39)	1,118	1.25 (0.91 ~ 1.71)	0.173	1.22 (0.88 ~ 1.68)	0.229	1.22 (0.88 ~ 1.69)	0.242
Q3(1.40–1.99)	1,115	1.26 (0.92 ~ 1.73)	0.151	1.23 (0.89 ~ 1.69)	0.214	1.22 (0.86 ~ 1.73)	0.266
Q4(>1.99)	1,114	1.58 (1.13 ~ 2.2)	0.007	1.49 (1.06 ~ 2.09)	0.021	1.51 (1.01 ~ 2.24)	0.044
Trend test	4,453	1.15 (1.03 ~ 1.27)	0.01	1.13 (1.01 ~ 1.25)	0.027	1.13 (1 ~ 1.28)	0.06

Q, quartiles; OR, odds ratio; CI, confidence interval; Ref: reference. Model 1 adjusted for sociodemographic (age, race/ethnicity, education level, family income and marital status). Model 2 was adjusted for Model 1 plus BMI, smoking status, vigorous activity, moderate activity, alcohol consumption, use of birth control pills, high blood pressure, diabetes, coronary heart disease and chronic bronchitis. Model 3 was adjusted for Model 2 plus calorie consumption, total fat consumption, total cholesterol consumption and dietary supplements taken.

### Stratified analyses based on additional variables

**Figure 2:**
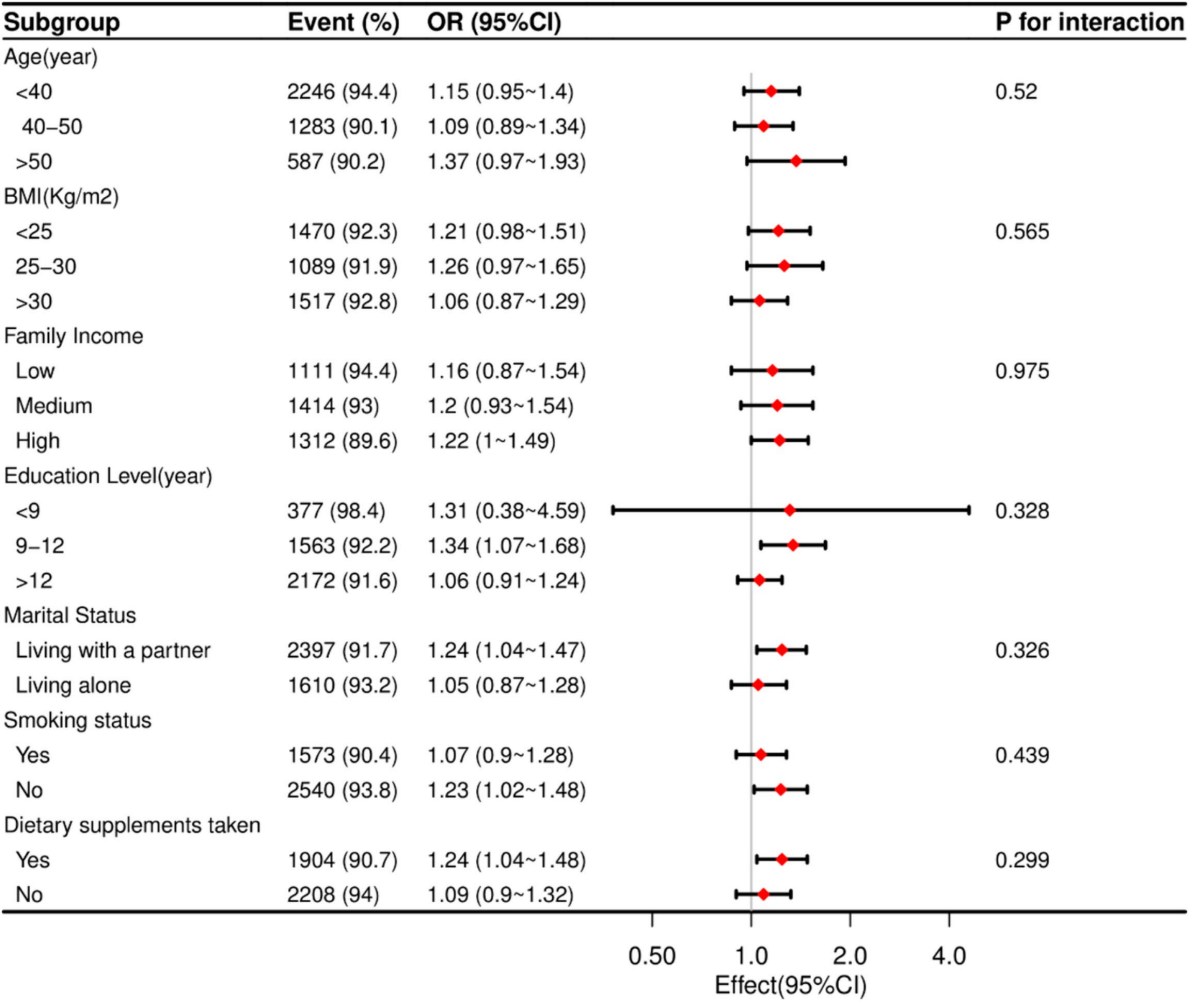
The relationship between dietary vitamin B6 intake and endometriosis according to basic features. Except for the stratification component itself, each stratification factor was adjusted for all other variables (age, marital status, race/ethnicity, education level, family income, BMI, vigorous activity, moderate activity, dietary supplements taken and smoking status).

In a thorough examination of various subgroups, stratified analyses were conducted to assess potential effect modifications on the relationship between dietary vitamin B6 intake and endometriosis (refer to Figure 2). Notably, no significant interactions were identified in any subgroups, whether stratified by age, BMI, marital status, family income, smoking status, education level, or dietary supplements taken. These results suggest that the observed association between dietary vitamin B6 intake and endometriosis remains consistent across diverse demographic and lifestyle factors, reinforcing the robustness of the findings.

### Sensitivity analysis

Furthermore, after excluding individuals with extreme energy intake, the dataset comprised 4,375 individuals, and the association between dietary vitamin B6 intake and endometriosis remained robust. Compared to individuals with lower vitamin B6 consumption (<0.96 mg/day), and after adjustments for age, race, education level, smoking status, dietary supplements taken, vigorous activity, moderate activity, marital status, family income, and BMI, the adjusted odds ratio (OR) values for dietary vitamin B6 intake and endometriosis in Q2 (0.96–1.39 mg/day), Q3 (1.40–2.05 mg/day), and Q4 (>2.05 mg/day) were 1.26 (95% CI: 0.91–1.73, *p* = 0.16), 1.27 (95% CI: 0.92–1.75, *p* = 0.15), and 1.61 (95% CI: 1.14–2.26, *p* = 0.01), respectively (Supplementary Table S1).

## Discussion

In this comprehensive cross-sectional study involving American adults, a noteworthy and previously unexplored positive correlation between endometriosis and dietary vitamin B6 intake was identified. Upon stratified analysis, these associations were found to be more significant among patients from high-income families, with 9–12 years of education, living with a partner, non-smokers, and those eligible for dietary supplements. Endometriosis has been frequently linked to inflammation (28), and evidence from studies suggested that women who have the disease exhibited greater levels of both systemic and localized inflammation. This led to the hypothesis that antioxidants, including vitamin B6, may contribute to the modulation of endometriosis, given its roles as an antioxidant regulator. This finding suggested a possible association between dietary factors and endometriosis, indicating a need for further research to explore this relationship in detail.

Given the observed correlation, it is essential to delve deeper into the specific biochemical mechanisms by which Vitamin B6 may influence endometriosis, particularly through its antioxidant and anti-inflammatory effects. Vitamin B6, present in forms such as pyridoxine, pyridoxal, and pyridoxamine, along with their phosphorylated derivatives, is known for its potent antioxidant properties, particularly in scavenging reactive oxygen species (ROS) (7, 29). This function is crucial in managing conditions like endometriosis, characterized by chronic inflammation and oxidative stress. The active form, pyridoxal 5′-phosphate (PLP), is vital in the transsulfuration pathway, where it helps convert homocysteine into cysteine, a precursor for the synthesis of glutathione (30). Glutathione, a key antioxidant, neutralizes ROS, thus protecting cells from oxidative damage and maintaining a balanced redox state (31, 32).

In endometriosis, elevated ROS levels lead to cellular damage, such as lipid peroxidation, DNA damage, and protein oxidation, which exacerbate inflammation in endometrial tissues (31). Vitamin B6 contributes to reducing oxidative stress and inflammation by promoting glutathione production, thereby mitigating these damaging effects (32, 33). This antioxidant defense and regulation of inflammation are crucial in controlling the progression and symptoms of endometriosis.

Additionally, research indicates that sufficient levels of Vitamin B6 can lower pro-inflammatory cytokines like IL-6 and TNF-*α*, which are significant mediators in inflammatory responses and are linked to worsening chronic inflammatory conditions (34). This anti-inflammatory property of Vitamin B6 suggests its potential therapeutic use in not only endometriosis but also other inflammatory diseases, such as rheumatoid arthritis and cardiovascular disorders (35).

Moreover, the enhancement of glutathione activity by Vitamin B6 underscores its role in maintaining cellular redox balance, which is essential for preventing oxidative damage and managing inflammation in chronic diseases (36). Studies have shown that Vitamin B6 supplementation can improve markers of oxidative stress and reduce inflammation, demonstrating its protective effects against cellular damage (37). In summary, Vitamin B6 is integral in supporting glutathione synthesis and modulating inflammatory responses, offering potential benefits in managing chronic inflammatory diseases like endometriosis. Further research is necessary to fully understand these mechanisms and develop effective therapeutic applications.

Our study possessed several strengths. Firstly, it was the first research explicitly examining the association between dietary vitamin B6 consumption and endometriosis. We identified a positive correlation between dietary vitamin B6 intake and endometriosis, and these results remained robust after conducting multiple regression and sensitivity analyses. Our study had several limitations, though. Firstly, because of the cross-sectional design, we were unable to prove directionality or causation. The existence of unmeasured variables may have added confounding effects even when possible confounders were carefully adjusted for in the logistic regression model. Interestingly, our model included several dietary parameters in an attempt to reduce confounding effects. Secondly, it was still challenging to measure the complete body’s vitamin B6 status precisely. We had inherent constraints in measuring vitamin B6 consumption because we relied on dietary questionnaires and 24-h memory. Because self-reported dietary data were prone to recall bias, they could not provide accurate assessments of a person’s overall vitamin B6 level. The accuracy of these assessments could be improved in the future by adopting more advanced approaches for assessing vitamin B6 levels. Thirdly, the study’s unique emphasis on citizens of the US meant that conclusions should be extrapolated with caution to other populations. Our results may not have been as broadly applicable as they could be due to the distinctive lifestyle and demographic characteristics of the US. Therefore, to confirm and broaden the applicability of our findings, carefully planned multicenter controlled studies including a variety of populations are essential. In conclusion, although our investigation illuminated the complex correlation between vitamin B6 intake in the diet and endometriosis, the limitations we found emphasized the necessity for additional study to fully comprehend this link and its wider consequences.

## Conclusion

In this study, we identified a positive association between dietary vitamin B6 intake and the risk of endometriosis among American women aged 20 to 54 years. The findings suggest that higher dietary intake of vitamin B6 may be linked to an increased risk of developing endometriosis, particularly in women with specific sociodemographic characteristics. This study adds to the growing body of evidence highlighting the potential role of diet in the pathogenesis of endometriosis. However, given the observational nature of this study, further research is warranted to explore the underlying mechanisms and to establish causal relationships. Future studies should also consider a broader range of populations and utilize longitudinal designs to validate these findings and examine the long-term effects of dietary vitamin B6 on endometriosis risk.

## Data Availability

The original contributions presented in the study are included in the article/Supplementary material, further inquiries can be directed to the corresponding author.
