# Supplementary material for: Association between dietary vitamin B6 intake and endometriosis risk: evidence from the national health and nutrition examination survey
Source: Front Nutr. 2024 Oct 3;11:1407099. doi: 10.3389/fnut.2024.1407099 (PMC11483862; doi:10.3389/fnut.2024.1407099)
Supplement: Supplementary file 1 [file Table_1.DOCX]

Supplementary Table S1. sensitivity analysis

| Variable |  |  | OR (95%CI) |  |  |  |  |
| --- | --- | --- | --- | --- | --- | --- | --- |
|  | total | Model 1 | P_value | Model 2 | P_value | Model 3 | P_value |
| Dietary vitamin B6 intake(mg/d) | 4375 | 1.17 (1.03~1.34) | 0.019 | 1.2 (1.04~1.38) | 0.013 | 1.2 (1.04~1.38) | 0.012 |
| Q1(<0.957) | 1094 | 1(Ref) |  | 1(Ref) |  | 1(Ref) |  |
| Q2(0.958-1.393) | 1092 | 1.15 (0.85~1.56) | 0.362 | 1.25 (0.91~1.72) | 0.171 | 1.26 (0.91~1.73) | 0.159 |
| Q3(1.394-2.053) | 1095 | 1.23 (0.91~1.67) | 0.183 | 1.27 (0.92~1.75) | 0.138 | 1.27 (0.92~1.75) | 0.145 |
| Q4(>2.054) | 1094 | 1.46 (1.06~2.01) | 0.02 | 1.59 (1.14~2.23) | 0.007 | 1.61 (1.14~2.26) | 0.006 |
| Trend test | 4375 | 1.13 (1.02~1.25) | 0.019 | 1.15 (1.04~1.28) | 0.009 | 1.15 (1.04~1.28) | 0.009 |

Q, quartiles; OR, odds ratio; CI, confidence interval; Ref: reference.

Model 1 was a crude model.

Model 2 was adjusted for sociodemographic (age, race/ethnicity, education level, marital status, family income and BMI)

Model 3 was adjusted for Model 2 plus vigorous activity, moderate activity, dietary supplements taken and smoking status.
